# Shear Rheology of Unentangled and Marginally Entangled Ring Polymer Melts from Large-Scale Nonequilibrium Molecular Dynamics Simulations

**DOI:** 10.3390/polym11071194

**Published:** 2019-07-17

**Authors:** Alexandros J. Tsamopoulos, Anna F. Katsarou, Dimitrios G. Tsalikis, Vlasis G. Mavrantzas

**Affiliations:** 1 Department of Chemical Engineering, University of Patras and FORTH-ICE/HT, GR 26504 Patras, Greece; 2 Particle Technology Laboratory, Department of Mechanical and Process Engineering, ETH Zürich, CH-8092 Zürich, Switzerland

**Keywords:** ring polymers, nonequilibrium simulation, polymer rheology

## Abstract

We present results for the steady state shear rheology of non-concatenated, unentangled and marginally entangled ring poly(ethylene oxide) (PEO) melts from detailed, atomistic nonequilibrium molecular dynamics (NEMD) simulations, and compare them to the behavior of the corresponding linear melts. The applied flow field spans a wide range of shear rates, from the linear (Newtonian) to the highly non-linear (described by a power law) regime. For all melts studied, rings are found to exhibit shear thinning but to a lesser degree compared to linear counterparts, mostly due to their reduced deformability and stronger resistance to alignment in the direction of flow. These features are attributed to the more compact structure of ring molecules compared to linear chains; the latter are capable of adopting wider and more open conformations even under shear due to the freedom provided by the free ends. Similar to linear melts, rings also exhibit a first and a second normal stress coefficient; the latter is negative. The ratio of the magnitude of the two coefficients remains practically constant with shear rate and is systematically higher than the corresponding one for linear melts. Emphasis was also given to the statistics of terminal (re-orientational) relaxation times which we computed by analyzing all chains in the simulated systems one by one; it was demonstrated that long time dynamics are strongly heterogeneous both for rings and (especially) linears. Repeating the analysis under flow conditions, and as expected, we found that the applied flow field significantly suppresses dynamic heterogeneity, especially for high shear rates well beyond the Newtonian plateau. Finally, a detailed geometrical analysis revealed that the average population of ring–ring threading events in the longest melt studied here (the PEO-5k ring) remains practically unaffected by the imposed flow rate even at strong shear rates, except for multi-threadings which disappear. To further analyze this peculiar and rather unexpected effect, we computed the corresponding survival times and penetration lengths, and found that the overwhelming majority of threadings under shear are extremely weak constraints, as they are characterized by very small penetration lengths, thus also by short survival times. They are expected therefore to play only a minor (if any) role on chain dynamics.

## 1. Introduction

Due to their molecular architecture (absence of free ends, looped structure), ring polymers exhibit distinct dynamics, and rheological behavior that differs substantially from that of linear analogues. The work of Kapnistos et al. [1] revealed that highly purified entangled ring melts do not exhibit the well-known plateau modulus, but an extended power law regime where the shear modulus of relaxation *G*(*t*) scales with time *t* as *G*(*t*) ~ *t*^0.4^ followed by the terminal relaxation zone. The latter is faster than in linear counterparts and obeys the expected exponential decay with time [2], which cannot be fully captured by theoretical models available today. This terminal relaxation appears to be characterized by a slow mode linked either with linear impurities that are not accounted for in theoretical models [1,3] or, as suggested by atomistic simulations, by ring–ring threading events [4,5]. Although these observations are supported by experiments [6,7] and simulations with coarse-grained models [8,9], further studies are definitely needed to fully elucidate and quantify the microscopic mechanisms responsible for the slow terminal relaxation exhibited by polymer rings.

In addition to *G*(*t*), the closed structure of rings can strongly influence their linear rheology. Experimental measurements [6] have shown that the zero shear rate viscosity $\eta_{0}$ of ring polymer melts scales with the degree of polymerization or molecular length *N* as $\eta_{0}{\sim N}^{b}$, with *b* approximately equal to 1 for a range of molar masses that extend up to $M_{w}\approx {5\;M}_{e}$ ($M_{e}$ is the characteristic entanglement molecular weight of the linear analogue). This is consistent with the corresponding Rouse theory for rings (the so-called Rouse ring model) according to which b=1 [10]. In contrast, we know that the viscosity of the corresponding linear analogues departs from the Rouse behavior (to enter reptation dynamics) much earlier (e.g., for $M_{w}{>2\;M}_{e}$). For molecular weights *M* above 5 *M*_e_, the power law dependence of *η*_0_ on *M*_w_ for rings becomes stronger. For example, the viscosity data of Doi et al. [6] suggest that for entangled ring melts b=2.4±0.1. This is in contrast to the prediction of the lattice animal model according to which b=3/2 [1] or to the recent fractal loopy globule model for which b=1.33 [11]. On the other hand, several recent molecular dynamics (MD) simulations based either on coarse-grained [8] or atomistic [12] models report values for the exponent *b* that lie in the region of 1.4–1.7. Further studies are thus needed to also elucidate this issue.

As far as the non-linear rheology of rings is concerned, this differs substantially from that of their linear analogues. Recent experiments with marginally entangled (*Z* = 5) ring polystyrene (PS) melts [13] have shown that rings also present a shear thinning behavior similar to linear melts, but a weaker one due to their smaller deformability by the flow. In particular, the rate of decay of viscosity in the non-linear regime has been found to be similar to that of unentangled linear melts [13]. An atomistic nonequilibrium molecular dynamics (NEMD) simulation with marginally entangled ring and linear polyethylene (PE) melts has reported similar findings [14]. 

As far as the transient shear rheology of ring melts is concerned, this is a field that to a large degree, still remains unexplored. To our knowledge, only the experimental work of Yan et al. [13] has addressed the time evolution of rheological material functions in shear for rings. Based on the transient shear viscosity *η*^+^ measurements of Yan et al. [13], rings exhibit similar qualitative behavior with linear chains. In particular, the *η*^+^ data collapse at low times to define what we call the linear viscoelastic envelope, followed by the characteristic overshoot maxima before steady-state values are reached. However, the maxima for the rings are smaller than for the linears. Moreover, the well-known undershoot succeeding the stress overshoot at strong shear rates typically observed for entangled linear melts is not observed for rings. Overall, and despite recent advances in purification techniques, the fundamental understanding of the linear and non-linear shear rheology of rings remains still a challenging issue [1,13].

In addition to experimental studies, computer simulations have also contributed substantially to our fundamental understanding of ring melt dynamics and rheology in recent years [8,9,10,12,14,15,16,17,18,19]. We can mention, for example, the work of Halverson et al. [8] who carried out coarse-grained equilibrium MD simulations of fully entangled pure ring and linear melts and showed that entangled rings do not exhibit a plateau in the stress relaxation modulus, in accordance with experiments [1], and are characterized by a zero shear rate viscosity that scales rather weakly with chain length ($\eta_{0}{\;\sim \;N}^{1.4\pm 0.2}$) compared to what is predicted by the reptation theory for linear melts ($\eta_{0}{\;\sim \;N}^{3.4\pm 0.2}$). In a more recent work, Yoon et al. [14] conducted atomistic NEMD simulations in shear and planar elongational flow with unentangled and marginally entangled pure ring and pure linear PE melts, and found that rings exhibit shear thinning but to a smaller degree compared to linears. In addition, their simulation results for the response of chain size to the applied flow field showed that rings exhibit a stronger resistance and deform less by the flow than linear polymers.

Our goal in the present work is to further contribute to our understanding of the shear rheology of polymer rings by carrying out a systematic study of their steady state rheological properties for sizes that span the regime of molecular weights from unentangled (*M*_w_ < *M*_e_) up to fully entangled ($M_{w\;}\geq {\;10\;M}_{e}$), which is currently missing. As a first step to this direction, we report here results from a detailed atomistic NEMD simulation study addressing well-characterized poly(ethylene oxide) (PEO) melts in this crossover regime. We chose to work with PEO because: (a) there exist in the literature several experimental data sets for its dynamics and rheology with which we can directly compare simulation findings and validate the accuracy of the obtained results, and (b) recent equilibrium MD simulations with the force field of Fischer et al. [20,21] yielded results for its equilibrium conformation and dynamics in excellent agreement with state-of-the-art experimental measurements [19]. The NEMD simulation results reported here have been obtained with the same force field and, as we will see, this will be reflected both in the quality of the simulation predictions and in their comparison with available experimental data. To compare with the corresponding rheology of linear melts, we also carried out NEMD simulations for the corresponding linear PEO melts. In all cases, the NEMD simulations were executed with very large simulation cells (containing in some cases up to two million interacting units) to ensure complete absence of system size effects and to add to the accuracy and reliability of the predicted rheological and conformational behavior.

This paper is organized as follows. In Section 2 we present some technical details concerning the simulation methodology, the atomistic force field employed in the MD and NEMD simulations, and the PEO systems studied. Our NEMD results for the rheological (shear viscosity and first and second normal stress coefficients), conformational (components of the radius-of-gyration tensor and alignment angle), relaxational (histograms of characteristic re-orientational relaxation times) and topological (density of entanglement kinks in the case of linears and percentage of threading events per molecule in the case of rings) are reported and discussed in detail in Section 3. Finally, in Section 4 we report the major findings of this work together with a brief outline of future plans and directions.

## 2. Systems Studied and Simulation Details

We have focused on melts of ring and linear PEO chains with the chemical structure −CH_2_−O−(CH_2_−CH_2_−O)_N_−CH_2_− for rings and CH_3_−O−(CH_2_−CH_2_−O)_N_−CH_3_ for linear chains, where *N* denotes the number of monomers per molecule (or, equivalently, the degree of polymerization). For both types of melts, we have considered three different chain sizes characterized by *N* = 29, 40 and 120. The entanglement molecular weight *M*_e_ of linear PEO is *M*_e_ = 2020 g/mol, which corresponds to *N*_e_ = 46. We understand then, that the linear systems with *N* = 29 and *N* = 40 are unentangled while the system with *N* = 120 is marginally entangled characterized by approximately *Z* = 2.5 entanglements per chain. The corresponding molecular weights (they differ slightly between ring and linear chains) are 1322, 1846 and 5326 g/mol. In the following, we will refer to the three systems as PEO-1k, PEO-2k and PEO-5k, respectively. The ring melts will be further denoted as R-1k, R-2k and R-5k, while the linear ones as L-1k, L-2k and L-5k.

For the NEMD simulations, we constructed very large rectangular simulation cells, significantly enlarged in the direction of flow (*x* direction) with the corresponding value *L_x_* of the simulation cell exceeding 170 nm in all cases (see Table 1). For example, for the longest ring melt addressed here (R-5k), the mean radius of gyration $R_{g}^{\mathrm{eq}}\;\left(={\langle R_{g,\mathrm{eq}}^{2}\rangle }^{\frac{1}{2}}\right)$ at equilibrium is $R_{g}^{\mathrm{eq}}$ = 18 ± 1 Å while the magnitude of the maximum diameter vector ($R_{d}^{\max}$) corresponding to a fully extended ring conformation is $R_{d}^{\max}$ ≈ 220 Å. This $R_{d}^{\max}$ is estimated under the approximation that it is equal to one half the fully extended end-to-end length of the linear analogue, $R_{d}^{\max}=0.5\;R_{\mathrm{ee}}^{\max}$. The dimensions of the corresponding NEMD simulation cell were chosen to be equal to (207 nm) × (11 nm) × (11 nm) in the *x*, *y* and *z* directions, respectively. This indicates that the simulation cell was ~9.4 times larger than $R_{d}^{\max}$ in the direction of flow and ~6.0 times larger than $R_{g}^{\mathrm{eq}}$ in the other two directions, implying that finite system size effects are kept to a minimum.

To generate fully relaxed structures for all systems studied, prior to the NEMD simulations, the systems were subjected to exhaustive (on the order of several microseconds) equilibrium MD simulations in the NPT statistical ensemble at temperature *T* = 363 K and pressure *P* = 1 atm with the force field of Fischer et al. [20,21] using GROMACS [22]. Fully relaxed configurations from these MD simulations were used as input in the subsequent nonequilibrium (flow) simulations under shear. The NEMD simulations were carried out with the LAMMPS software [23] in the NVT ensemble at *T* = 363 K using the p-SLLOD equations of motion [24,25] and the Nosé–Hoover thermostat [26,27,28] to control the temperature. The set of microscopic p-SLLOD equations of motion reads:

(1) $${\dot{q}}_{ia}=\frac{p_{ia}}{m_{ia}}+q_{ia}\cdot \nabla u$$

(2) $${\dot{p}}_{ia}=F_{ia}-p_{ia}\cdot \nabla u-m_{ia}q_{ia}\cdot \nabla u\cdot \nabla u-\frac{p_{\zeta }}{Q}p_{ia}$$

(3) $$\dot{\zeta }=\frac{p_{\zeta }}{Q}$$

(4) $${\dot{p}}_{\zeta }=\sum_{i}\sum_{a}\frac{p_{ia}^{2}}{m_{ia}}-Dnk_{B}T$$

where **p***_ia_*, **q***_ia_* and **F***_ia_* are the momentum, position and force vectors of atom *a* in molecule *i*, of mass *m_ia_*. In the above equations, *n* denotes the total number of atoms, *T* the absolute temperature, *k*_B_ the Boltzmann constant, and *D* the space dimensionality (in our case, *D* = 3). Also, *ζ* and *p*_ζ_ are the coordinate- and momentum-like variables of the Nosé–Hoover thermostat, ${Q=Dnk}_{B}{T\tau }^{2}$ denotes the mass parameter of the thermostat, and ∇u represents the velocity gradient tensor. According to Equations (1)–(4), flow in our work is imposed at the level of the microscopic equations of motion through the relevant velocity gradient tensor. In the case of shear flow, ∇u has the form:

(5) $$\nabla u=[\begin{array}{ccc} 0 & 0 & 0\\ \dot{\gamma } & 0 & 0\\ 0 & 0 & 0 \end{array}]$$

with $\dot{\gamma }$ representing the imposed strain rate. Note that in the case of shear, the term $m_{ia}q_{ia}\cdot \nabla u\cdot \nabla u$ in the momentum equation, Equation (2), vanishes. In conjunction with the Lees–Edwards [29] boundary conditions to prevent the periodic boundary conditions (PBCs) from interfering with particle trajectories, the p-SLLOD equations of motion generate the correct velocity gradient [30]. 

For the numerical integration of the equations of motion we made use of the reversible Reference System Propagator Algorithm (r-RESPA) [31], with three different time steps: (a) a large one (dt = 5 fs) for the integration of the slowest varying forces arising from electrostatic interactions at long interatomic distances; (b) an intermediate one (dt = 2.5 fs) for the integration of forces associated with electrostatic interactions at short distances and Lennard–Jones contributions, and of the terms referring to the Nosé–Hoover thermostat and the flow field; and (c) a small one (dt = 1.25 fs) for the integration of the fast-varying forces corresponding to all bonded interactions. The above scheme was repeatedly and thoroughly validated by carrying out a series of test NEMD simulations at various strain rates where the p-SLLOD equations of motion were integrated with the velocity-Verlet algorithm using a single, small time step (dt = 1 fs) for all forces. The results of the proposed multiple time step r-RESPA scheme were found to be statistically equivalent to those from the short time step velocity-Verlet scheme. We also report that, occasionally, we had to reduce the large time step to 2 fs in some of our simulations in the nonlinear regime (corresponding to *Wi* > 25) to ensure that electrostatic interactions at long interatomic separations were computed absolutely accurately. 

In our study, a broad range of shear rates were employed to investigate the rheological behavior of ring melts. At *T* = 363 K, the relaxation time characterizing the equilibrium dynamics of the longest model system studied here (linear PEO-5k) is a few hundreds of nanoseconds [12,19]. At sufficiently low shear rates (*Wi* ≤ 1) where the effect of flow on chain rotation is relatively small, the relaxation time of the simulated systems is expected to be almost equal to the equilibrium relaxation time [32]. Therefore, to obtain steady-state behavior and extract accurate predictions of the rheological material functions of interest and of the conformational and terminal relaxation properties, NEMD simulations on the order of several hundreds of nanoseconds up to a few microseconds were needed (see also Section 3.4). Clearly, without the help of r-RESPA, the NEMD simulations carried out here (particularly those at relatively weak flow rates corresponding to *Wi* < 10) for the longest melt addressed (PEO-5k) would not have been possible from a computational point of view. In contrast, as the shear rates increase (e.g., for *Wi* ≥ 10), the relaxation time decreases considerably as the applied flow enhances chain rotation and tumbling (see Section 3.4) [32]. As a result, the simulation time needed to obtain steady-state behavior and study the rheological response of the melt also decreases. This also explains why in our results below (Section 3) the statistical noise of the predicted simulation data for the viscometric and conformational properties of the simulated melts decreased as the shear rate increased. All simulation results reported and discussed in the subsequent sections of this paper have been obtained from the steady-state part of the atomistic NEMD trajectories where all quantities of interest were observed to fluctuate around constant average values.

## 3. Results

### 3.1. Rheological Properties

**Figure 1:**
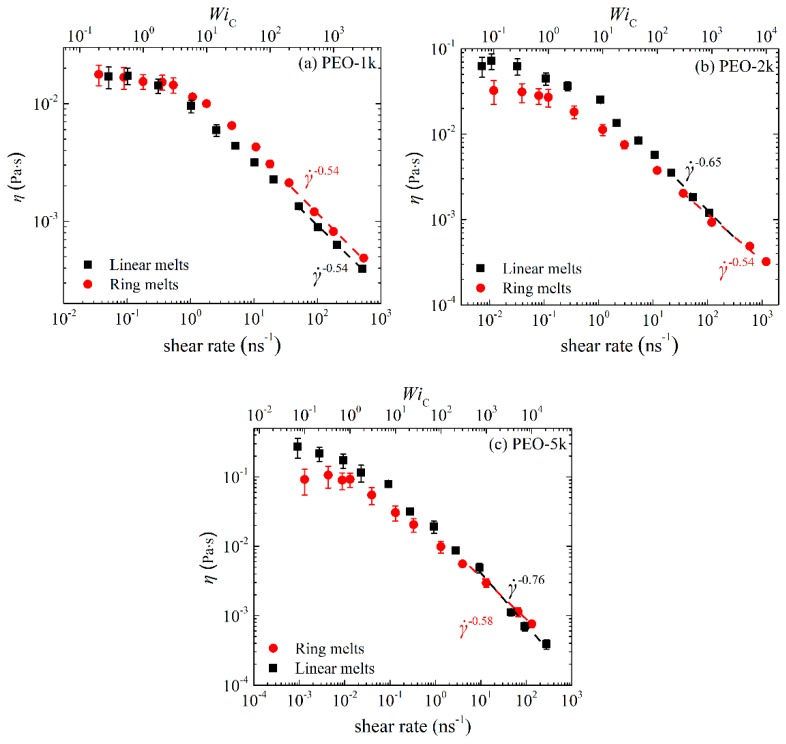
Steady-state shear viscosity *η* as a function of imposed strain rate or ${Wi}_{C}$ number for the simulated: (**a**) PEO-1k, (**b**) PEO-2k and (**c**) PEO-5k ring and linear melts.

Figure 1a–c display the steady-state state viscosity *η* as a function of the imposed strain rate $\dot{\gamma }$ for the simulated PEO-1k, PEO-2k and PEO-5k ring and linear melts. In each Figure, we have also added a second *x* axis at the upper part of the graphs indicating the corresponding Weissenberg number ${Wi}_{C}$ (subscript C coming from Cyclic) defined as ${Wi}_{C}=\tau_{R}^{\mathrm{ring}}\times \dot{\gamma }$, i.e., with respect to the ring orientational relaxation time $\tau_{R}^{\mathrm{ring}}$ under equilibrium conditions. Qualitatively, for all three different *M*_w_ ring PEO melts simulated, we observe the typical characteristic behavior of the shear viscosity with shear rate already known for linear polymer melts: (a) At low shear rates (${Wi}_{C}\leq 1$), the viscosity is practically constant defining what we know as the Newtonian plateau. (b) At higher shear rates, the viscosity starts decreasing, exhibiting what we know from the corresponding behavior of linear polymers as shear thinning. (c) At even higher shear rates, we enter the highly nonlinear regime where the viscosity drops rapidly with applied shear rate. It is interesting that for the PEO-1k system, ring and linear melts in the Newtonian regime exhibit similar viscosity values. On the other hand, for the PEO-2k and PEO-5k systems, the viscosity of the linear melt in the Newtonian regime is larger than that of the corresponding ring, which is in full agreement with experimental measurements [1,2,6,13,33] and previous simulation studies [8,12,14]. Overall, it appears that differences in the viscosity between ring and linear PEO melts in the Newtonian regime become more important as the *M*_w_ increases.

Given that the calculation of the stress tensor in atomistic NEMD simulations at very low shear rates (approximately below $1/\tau_{R}^{\mathrm{ring}}$) suffers from large statistical fluctuations, to compute the corresponding zero shear rate viscosity *η*_0_ we fit the simulation data to the Carreau Model [34,35]:

(6) $$\eta (\dot{\gamma })=\eta_{0}{\left[1+{\left(\lambda \dot{\gamma }\right)}^{2}\right]}^{-p}$$

where *p* is a characteristic exponent and *λ* a fitting parameter with units of time. All results derived from the Carreau model for *η*_0_, along with their standard deviation, are listed in Table 2. Very rough estimates of *η*_0_ were also obtained from the longest relaxation times for linears and rings $\tau_{1,L}$ (and $\tau_{2,R}$, respectively) as computed directly from the equilibrium MD simulations using the corresponding Rouse equations, namely $\eta_{0,L}=\left(\frac{\pi^{2}\rho RT\;}{12}\right)\tau_{1,L}$ and $\eta_{0,R}=\left(\frac{\pi^{2}\rho RT\;}{6}\right)\tau_{2,R}$; they are reported in the fourth and fifth column of Table 2. In the sixth and seventh column of Table 2, we report available experimental data for the zero-shear rate viscosity of PEO [12,19].

Using Equation (6) to compute *η*_0__,__L_ proved very efficient allowing us to reliably estimate the ratio $\eta_{0,L}/\eta_{0,R}$ of the zero-shear rate viscosity of the linear melt to the corresponding zero shear rate viscosity of the ring. As already mentioned, for the shortest melt examined (PEO-1k), the NEMD prediction for the viscosity ratio between linear and ring melt is $\eta_{0,L}/\eta_{0,R}=\;1.0\pm 0.1$. This observation agrees nicely with the experimental measurements of Nam et al. [36] for short ring and linear PEO melts at a lower temperature (*T* = 329 K) than in our NEMD simulations (*T* = 363 K), where the ratio $\eta_{0,L}/\eta_{0,R}$ was equal to 1 for melts with *M*_w_ ≈ 1500 g/mol. We remind the reader that according to the Rouse model the ratio $\eta_{0,L}/\eta_{0,R}$ is equal to 2 [10]. The reason for the deviation of the NEMD prediction from the Rouse model should be due to the excess free volume phenomena present in the linear melts due to chain ends, which accelerate chain dynamics and, in turn, cause the viscosity of the linear melt to decrease. Of course, as the chain length increases, these excess free volume phenomena become less and less important, thus we expect the ratio of the two viscosities to come closer to the value of 2.0 predicted by the Rouse theory. Indeed, for the PEO-2k melt, the corresponding ratio is $\eta_{0,L}/\eta_{0,R}=2.1\pm 0.1$, a result which is fully consistent with the Rouse model [10]. On the other hand, by further increasing *M*_w_, entanglements start developing between chains in the linear melt which restrict their dynamics; thus, now, we expect the viscosity of the linear melt to increase faster than the viscosity of the ring. Indeed, according to our NEMD simulations, for PEO-5k (a marginally entangled melt), $\eta_{0,L}/\eta_{0,R}=2.6\pm 0.1$. The fact that a change in the relaxation mechanism takes place as we cross over from PEO-2k to PEO-5k that cannot be accommodated by the Rouse model is also reflected in the unrealistically large value of the ratio $\eta_{0,L}/\eta_{0,R}$ (=8.5 ± 1), see data in the fourth and fifth column in Table 2 for PEO-5k, predicted by naïve application of the Rouse model equations on the basis of the computed chain orientational relaxation times for the ring and linear melt from the equilibrium MD data. Overall, we can say that, according to our NEMD simulations, the ratio $\eta_{0,L}/\eta_{0,R}$ for very short PEO melts (well below *M*_e_) starts from a value close to 1 and increases smoothly with *M*_w_, reflecting the faster increase of the viscosity of the corresponding linear melt as inter-chain entanglements start playing a role. Our NEMD data for *η*_0_ are also in very favorable agreement with experimentally measured data from Refs. [19,33].

In the non-linear regime, the viscosities of ring and linear melts behave qualitatively very similar for all chain lengths, but the degree of shear thinning in the linear melt is sharper compared to that in the corresponding ring melt, especially as the chain length increases. By fitting the viscosity curves with a power law of the form $\eta_{0}\sim {\dot{\gamma }}^{-b}$ we can extract the value of the exponent *b* quantifying the rate of shear thinning with applied shear rate. For the ring melts, the values obtained are: (a) *b* = 0.54 for R-1k, (b) *b* = 0.54 for R-2k, and (c) *b* = 0.58 for R-5k. For the linear melts, the corresponding values are: (a) *b* = 0.54 for L-1k, (b) *b* = 0.65 for L-2k, and (c) *b* = 0.76 for L-5k. We see that the shear thinning exponents for the linear melts increase more rapidly with chain size than the corresponding ones for the rings. This agrees with recent simulation [14] and experimental [13] studies, and is typically attributed (at least to a large extent) to the reduced deformability of ring chains [13,14] due to their closed-loop structure. For rings, it has been further argued that ring-specific dynamical mechanisms (including cooperative orientation of parallel segments or tumbling) can lead to molecular individualism, i.e., to a different behavior of different ring molecules in the melt [13,14]. We will come back to this issue later in Section 3.2 where we will examine the effect of flow on the rate of chain orientation of individual chains in the two types of melts (ring and linear).

**Figure 2:**
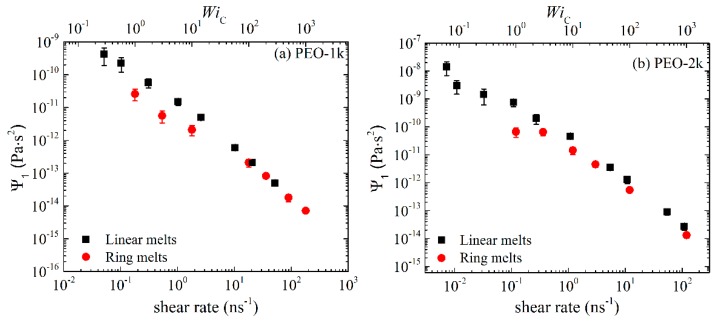

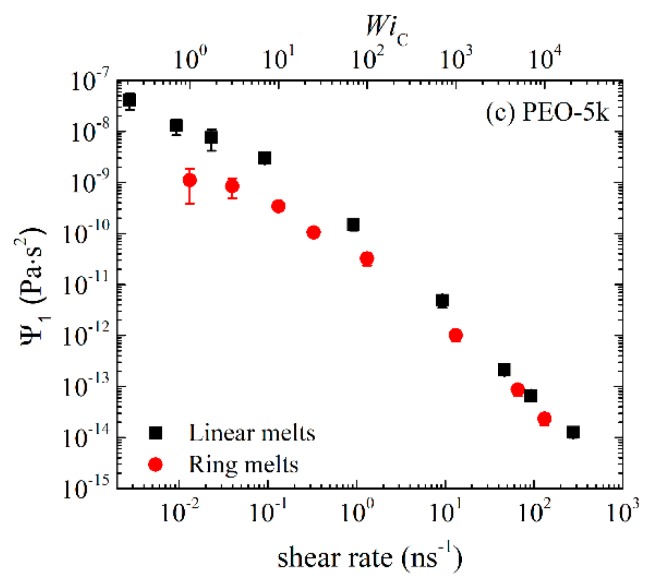
First normal stress coefficient Ψ_1_ as a function of imposed strain rate or ${Wi}_{C}$ number for the simulated: (**a**) PEO-1k, (**b**) PEO-2k and (**c**) PEO-5k ring and linear melts.

**Figure 3:**
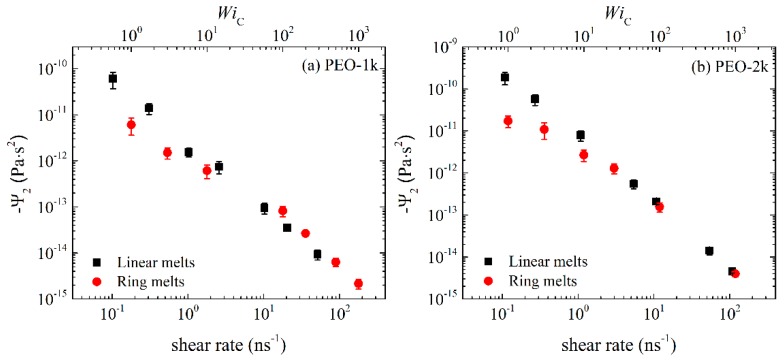

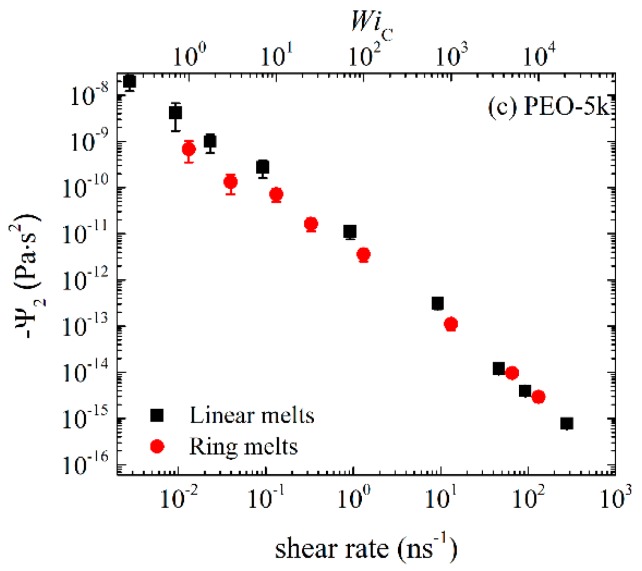
Second normal stress coefficient, −Ψ_2_, as a function of imposed strain rate or ${Wi}_{C}$ number for the simulated: (**a**) PEO-1k, (**b**) PEO-2k and (**c**) PEO-5k ring and linear melts.

In Figure 2 and Figure 3 we show the NEMD results for the first $\Psi_{1}\equiv (\sigma_{\mathrm{xx}}-\sigma_{\mathrm{yy}})/{\dot{\gamma }}^{2}$ and second $\Psi_{2}\equiv (\sigma_{\mathrm{xx}}-\sigma_{\mathrm{yy}})/{\dot{\gamma }}^{2}$ normal stress coefficient. We see that Ψ_1_ is positive and Ψ_2_ negative (this explains why in Figure 3, we plot −Ψ_2_), and that the magnitude of Ψ_2_ is much smaller than the magnitude of Ψ_1_. For weak-to-moderate shear rates (${Wi}_{C}<100$), ring melts exhibit smaller Ψ_1_ and −Ψ_2_ values compared to linear melts. For higher shear rates, both Ψ_1_ and −Ψ_2_ exhibit large power law regions, similar to those for $\eta (\dot{\gamma })$, decreasing by several orders of magnitude with shear rate. We also observe that the rate of decline of Ψ_1_ and −Ψ_2_ with $\dot{\gamma }$ is greater in the linear melts than in the rings. To quantify this difference, we fit the simulation data for Ψ_1_ and −Ψ_2_ with $\dot{\gamma }$ for ${Wi}_{C}<100$ with a power law of the form $\Psi_{1}\sim {\dot{\gamma }}^{-b_{1}}$ and $\Psi_{2}\sim {\dot{\gamma }}^{-b_{2}}$. For PEO-1k the two exponents are similar: *b*_1_ = 1.38 ± 0.05 and *b*_2_ = 1.21 ± 0.05 for the ring, and *b*_1_ = 1.38 ± 0.04 and *b*_2_ = 1.41 ± 0.05 for the linear melt. With increasing chain size, the differences in the power law exponents between ring and linear melts increase. Thus, for PEO-2k, *b*_1_ = 1.48 ± 0.04 and *b*_2_ = 1.44 ± 0.09 for the ring melt, which should be compared to *b*_1_ = 1.61 ± 0.03 and *b*_2_ = 1.63 ± 0.03 for the linear melt. For the marginally entangled PEO-5k, *b*_1_ = 1.55 ± 0.06 and *b*_2_ = 1.53 ± 0.03 for the ring melt, while *b*_1_ = 1.76 ± 0.05 and *b*_2_ = 1.75 ± 0.04 for the linear melt. Our simulation findings are in reasonable agreement with the results of [14] for marginally entangled (*Z* = 6) linear and ring PE melts. In particular, the Ψ_1_ and Ψ_2_ exponents reported in [14] are *b*_1_ = 1.45 ± 0.03 and *b*_2_ = 1.49 ± 0.11 for ring PE, which increased to *b*_1_ = 1.61 ± 0.03 and *b*_2_ = 1.67 ± 0.12 for linear PE. The finite extensibility of rings, due to their loopy geometry and more compact arrangement of monomers around their center of mass, should be considered as the main factor responsible for the weaker dependence of Ψ_1_ and Ψ_2_ on shear rate compared to linear melts. 

**Figure 4:**
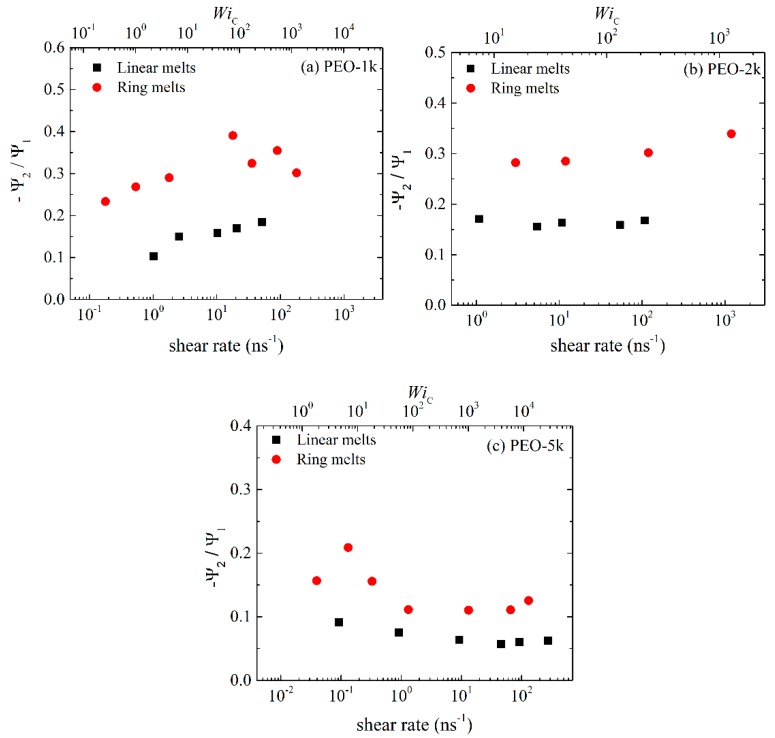
The ratio $-\Psi_{2}/\Psi_{1}$ as a function of applied shear rate or ${Wi}_{C}$ number for the simulated: (**a**) PEO-1k, (**b**) PEO-2k and (**c**) PEO-5k ring and linear melts.

Figure 4 presents the ratio $-\Psi_{2}/\Psi_{1}$ for ring and linear melts, for the three PEO systems studied. For all of them, rings exhibit larger $-\Psi_{2}/\Psi_{1}$ values compared to linear melts, which reflects again the reduced deformability of rings due to their closed loop architecture. As the molecular weight increases, for both linear and ring melts the ratio $-\Psi_{2}/\Psi_{1}$ decreases. Overall, and for the range of shear rates addressed in the present study, the ratio $-\Psi_{2}/\Psi_{1}$ for the ring melts ranges between 0.23 and 0.36 for R-1k, between 0.28 and 0.33 for R-2k, and between 0.11 and 0.21 for R-5k. For the linear melts, on the other hand, $-\Psi_{2}/\Psi_{1}$ ranges between 0.1 and 0.19 for L-1k, between 0.15 and 0.17 for L-2k, and between 0.05 and 0.10 for L-5k.

### 3.2. Conformational Properties

**Figure 5:**
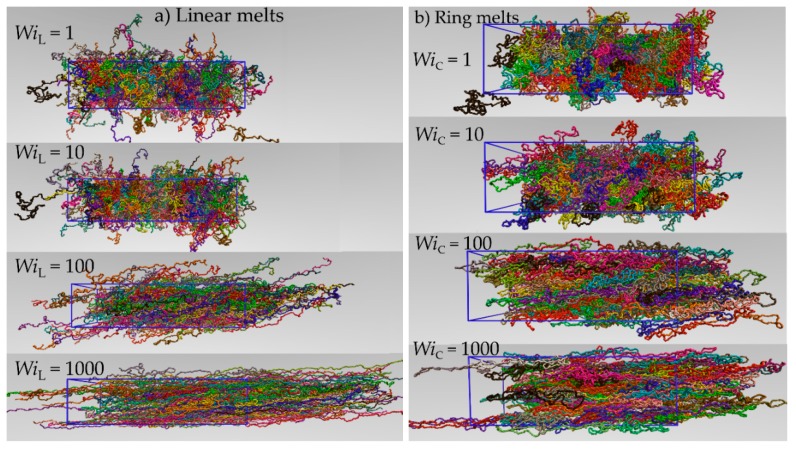
Representative atomistic configurations from the NEMD simulations with the linear (**a**) and ring (**b**) PEO-5k melt at various shear rates or ${Wi}_{C}$ numbers.

Figure 5a,b provide representative atomistic snapshots from the NEMD simulations with the marginally entangled PEO L-5k and R-5k melts, respectively, at four different shear rates corresponding to ${Wi}_{C}$ numbers equal to 1, 10, 100 and 1000. Admittedly, the applied flow has a strong effect on melt conformation; as shear rate increases, strong stretching and significant alignment of ring and linear PEO molecules along the flow (*x*-) direction are observed, causing significant deviations of their average shape from the equilibrium, isotropic configuration. To quantify this deviation, we have computed the average radius-of-gyration tensor as a function of applied flow. For a given chain, the *ab*-component of the second order radius-of-gyration tensor **G** is defined as

(7) $$G_{ab}=\overset{N}{\underset{i=1}{\sum }}\left(r_{a}^{i}-r_{a}^{\mathrm{com}}\right)\left(r_{b}^{i}-r_{b}^{\mathrm{com}}\right)$$

where *N* is the number of atoms per chain, $r^{i}$ and $r^{\mathrm{com}}$ denote the position vectors of atom *i* and of the center-of-mass (com) of the chain this atom belongs to, respectively, and the subscripts *a* and *b* indicate the space directions *a* and *b*, respectively.

**Figure 6:**
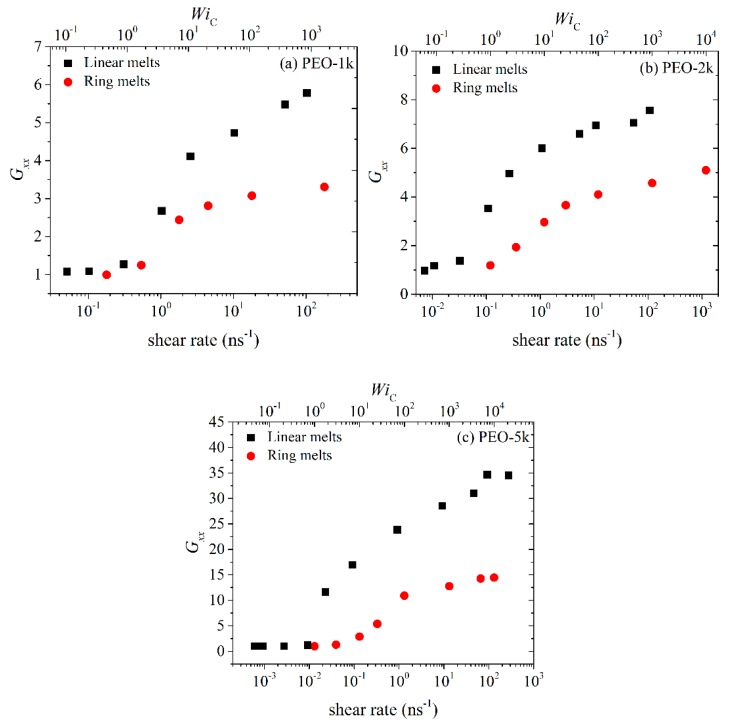
Variation of the *xx* component of the radius-of-gyration tensor with shear rate or ${Wi}_{C}$ number for the simulated: (**a**) PEO-1k, (**b**) PEO-2k and (**c**) PEO-5k ring and linear melts.

**Figure 7:**
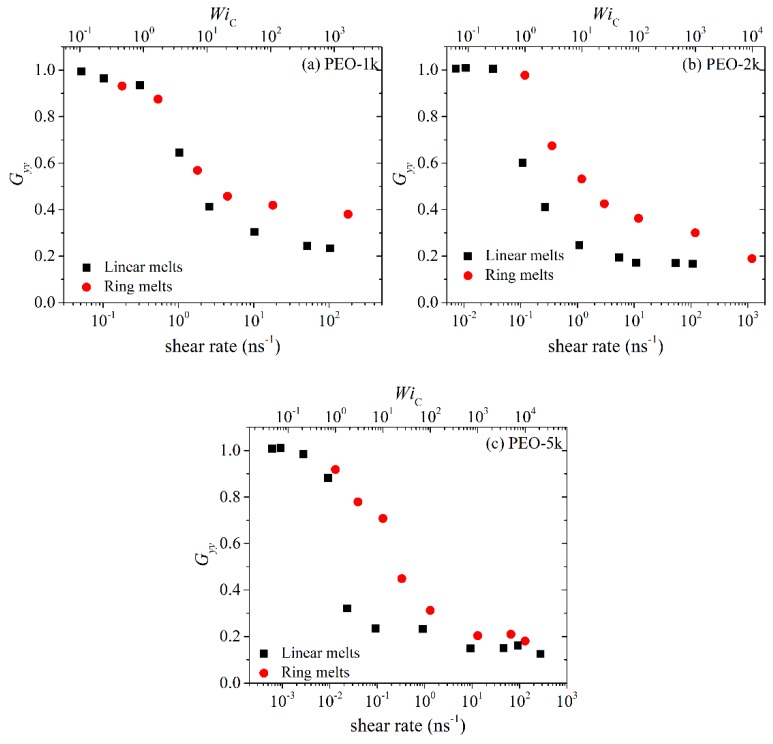
Same as with Figure 6 but for the *yy* component of the radius-of-gyration tensor.

**Figure 8:**
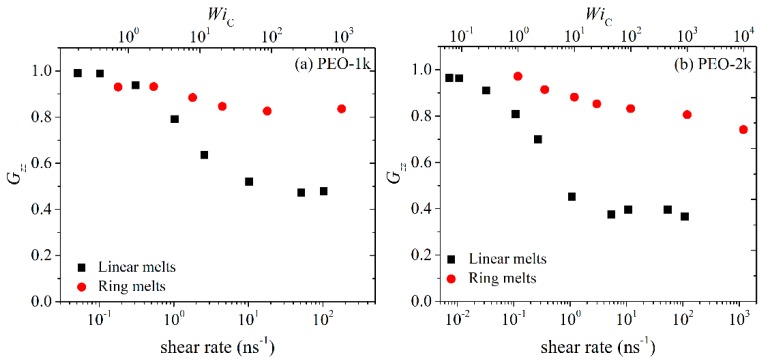

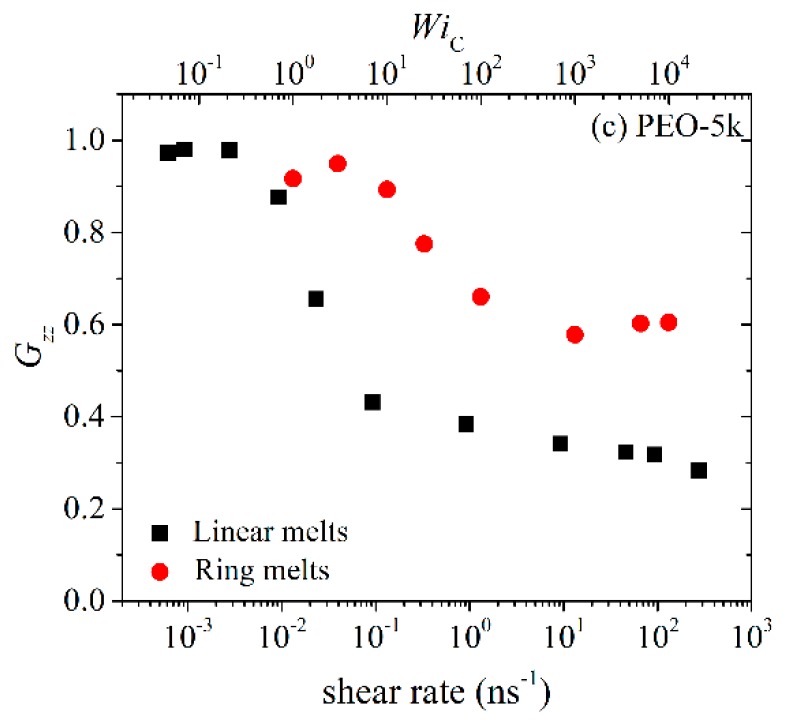
Same as with Figure 6 but for the *zz* component of the radius-of-gyration tensor.

**Figure 9:**
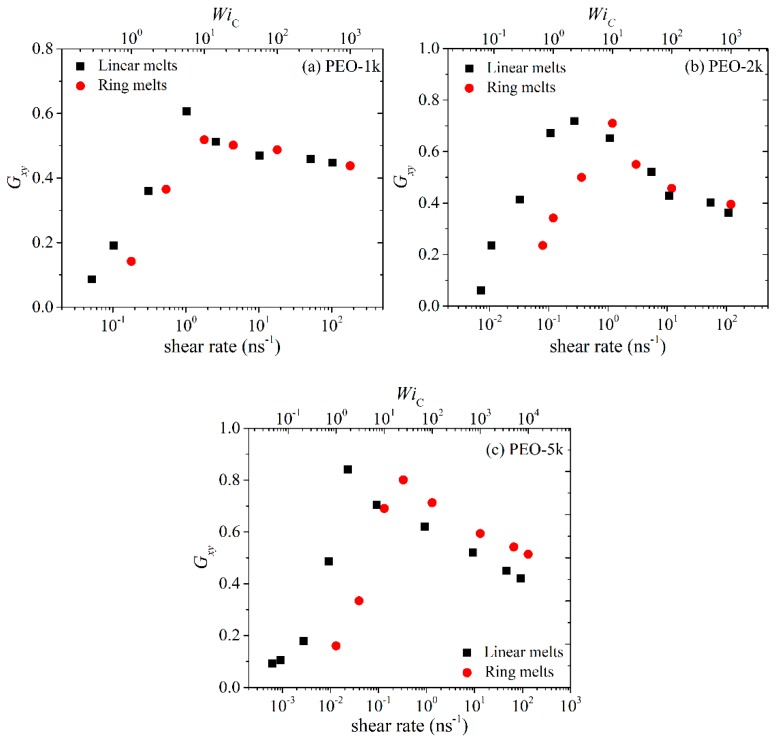
Same as with Figure 6 but for the *xy* component of the radius-of-gyration tensor.

In Figure 6, Figure 7, Figure 8 and Figure 9, we compare the *xx*, *yy*, *xy* and *zz* components of the radius-of-gyration tensor (*G_xx_*, *G_yy_*, *G_zz_* and *G_xy_*) between linear and ring melts. We focus our attention first on the *xx* component. At low shear rates (${Wi}_{C}<1$), the magnitude of *G_xx_* remains practically unaffected by the flow for both types of melts. At intermediate shear rates, chains in the melt deform and at the same time align in the direction of the flow, which causes a considerable increase in the value of *G_xx_*. At even higher shear rates, the rate of increase of *G_xx_* with shear rate declines and *G_xx_* approaches constant values which are, however, different between ring and linear melts. The fact that *G_xx_* assumes eventually constant values is due to two factors: (a) the finite extensibility of the simulated chains due to bond stretching and bond-bending interactions, and (b) chain rotation and tumbling due to the nature of the applied flow (shear) [32]. An interesting point to notice in the curves of Figure 6 is that for all three PEO melts studied, the ratio of the asymptotic *G_xx_* values between linear and ring melts is very similar and approximately equal to 2, which seems to suggest that, as far as their fully extended conformations are concerned, rings can be considered as linear chains of half the length.

On the other hand, the two other diagonal components *G_yy_* and *G_zz_* display exactly the opposite behavior. At low shear rates, their values remain unaffected by the imposed flow field; however, as the strength of the flow increases, both decrease considerably due to chain alignment in the direction of flow. It is also true that *G_yy_* decreases more rapidly than *G_zz_*, which should have been expected given that *z* is the neutral axis. As already mentioned, due to the nature of the applied flow, the average melt velocity varies along the *y*-axis, and this can cause chain rotation and tumbling (see below), which is another reason why *G_yy_* decreases faster than *G_zz_* at higher shear rates. As with many other (rheological and conformational) properties discussed so far, the values of *G_yy_* and *G_zz_* for rings are smaller than for linear melts, which is another manifestation of the more compact structure of cyclic molecules due to the more symmetric arrangement of atoms around their center-of-mass.

The shear rate dependence of the *xy* component of the radius-of-gyration tensor is presented in Figure 9. In all cases, *G_xy_* initially increases with applied shear rate, goes through a maximum at an intermediate value of shear rate, and then starts decreasing. It is known [32] that the overall change of *G_xy_* with respect to applied shear rate can be understood by considering two competing effects: (a) more open molecular conformations due to flow stretching in the *x*-direction and the spatial correlations between the *x* and *y* components of the chain end-to-end vector, leading to an increase in *G_xy_*, and (b) chain orientation along the flow direction, leading to a decrease in *G_xy_*. It is the competition between these two effects that gives rise to the maximum. Overall, and despite the obvious similarities in the overall qualitative behavior of *G_xy_* with shear rate, certain differences are observed. For example, for linear melts, *G_xy_* increases rapidly with shear rate even for small shear rates whereas for rings the increase is slower and is initiated at higher shear rates. This is another manifestation of stronger resistance to the applied flow, and can be explained again by the more compact structure of rings compared to linear melts which can attain more open and wider conformations. Similar results have been reported by Yoon et al. [14] for PE.

**Figure 10:**
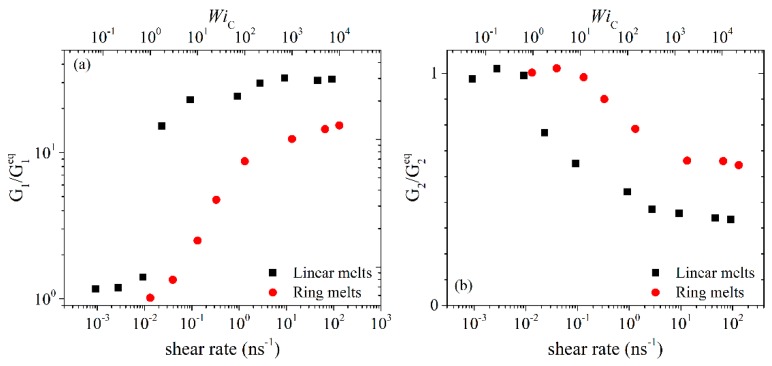

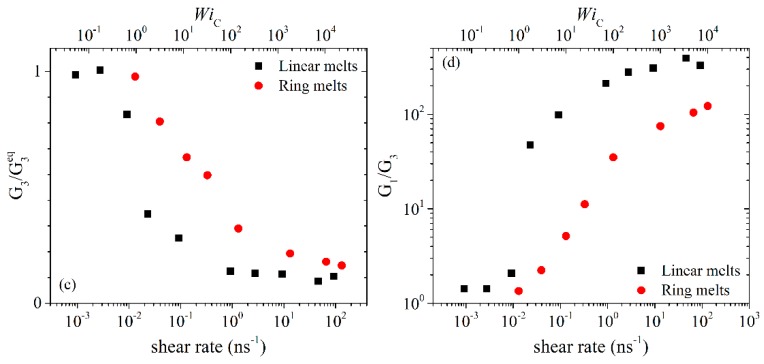
Variation of the three eigenvalues of the radius-of-gyration tensor with applied shear rate or ${Wi}_{C}$ number for the simulated ring and linear PEO-5k melts: (**a**) the largest eigenvalue *G*_1_, (**b**) the second largest eigenvalue *G*_2_, (**c**) the smallest eigenvalue *G*_3_, and (**d**) the ratio between *G*_1_ and *G*_3_.

To check how the intrinsic principal dimensions of the ring molecules change with applied flow, we have also calculated the eigenvalues (*G*_1_ > *G*_2_ > *G*_3_) of the radius-of-gyration tensor for all three different PEO melts simulated. The results found for the PEO-5k are reported in Figure 10. Also reported in Figure 10 is the ratio of the largest eigenvalue *G*_1_ with the smallest *G*_3_. Admittedly, the changes of the three eigenvalues with shear rate follow (to a large degree) the corresponding changes of the *xx*, *zz* and *yy* components of the radius-of-gyration tensor **G**.

**Figure 11:**
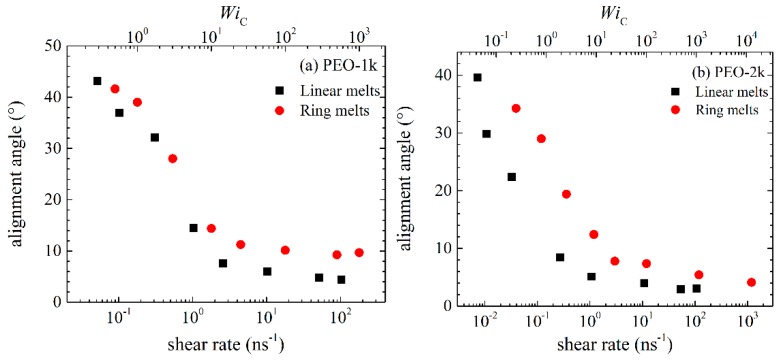

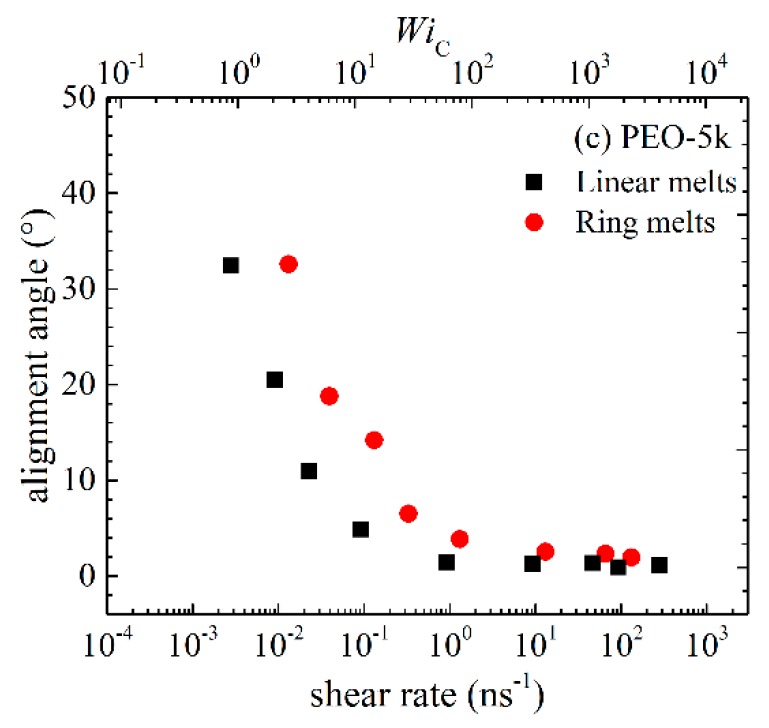
Same as with Figure 6 but for the alignment angle *θ*.

We have also studied the effect of the applied shear flow on chain alignment. To this, we calculated the alignment angle defined as

(8) $${2\theta =\tan}^{-1}\left(\frac{2\langle A_{xy}\rangle }{\langle A_{\mathrm{xx}}-A_{\mathrm{yy}}\rangle }\right)$$

where **A** denotes a second order tensor, e.g., the radius-of-gyration tensor, the stress tensor or the birefringence tensor. In the present work, we choose to work with the radius-of-gyration tensor **G**. Figure 11, then, shows how the alignment angle changes with applied shear rate between rings and linears. At low shear rates, *θ* tends to the value of 45° for both types of melts. The limiting value of 45° has already been observed in other simulation studies [14,32] and is also explained theoretically. Specifically, at low shear rates, the chains start to align, leading to a nonzero value of <*A_xy_*> but with negligible chain deformation (i.e., negligible normal stresses). At intermediate shear rates, the chains align in the flow direction and, also, stretch considerably; as a result, normal stresses develop in the melt, which in turn cause a steep rise in the value of *θ*. At high shear rates, chains reach their maximum allowable stretch, thus *θ* reaches a plateau value. Comparing the results between ring and linear melts at sufficiently high shear rates, we notice that rings are characterized by a lower degree of alignment. Once more, this demonstrates the stronger resistance of rings to the applied flow in comparison to linear chains [14] as a result of their shorter spatial extent due to their closed structure.

### 3.3. Terminal Relaxation

**Figure 12:**
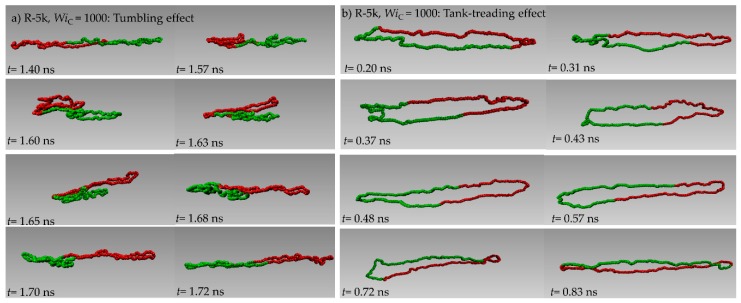
A series of instantaneous snapshots of two PEO-5k ring chains from the NEMD simulation at *Wi*_C_= 1000, undergoing: (**a**) tumbling (times between *t* = 1.40 ns and *t* = 1.72 ns), and (**b**) tank-treading (times between *t* = 0.20 ns and *t* = 0.83 ns). Chain segments have been colored red and green for better visualization of the two types of motion.

In the literature, it has been suggested that, similar to their linear counterparts, ring polymers can stretch, collapse, tumble and re-stretch when subjected to shear flow [37,38]. Moreover, due to their loopy structure, they can undergo tank-treading (i.e., a rotational motion where chain segments rotate along the deformed contour of the polymer) [37,38]. Simulations of dilute ring polymer solutions have shown [37,38] that at weak flow rates it is not easy to distinguish tumbling from tank-treading. In contrast, at strong flow rates, tumbling and tank-treading can occur independently. We observed similar tumbling and tank-treading dynamics in our NEMD simulations with the PEO melt systems addressed here. This is clearly discernible in Figure 12a,b, showing representative atomistic snapshots of two ring chains from the NEMD simulations with the R-5k melt under strong flow conditions (*Wi*_C_ = 1000) undergoing tumbling and tank-treading motion, respectively. The interested reader can also visualize the two types of motion for the chosen pairs of ring molecules in the two videos that we prepared and uploaded as Supplementary Material to this manuscript from the NEMD simulation with the R-5k melt at *Wi*_C_ = 1000.

Although polymers are characterized by a broad spectrum of characteristic times that span a wide range of scales, the most important one is the time associated with the relaxation of length scales on the order of the entire chain, defining what we call orientational or terminal relaxation. For linear melts, terminal relaxation is quantified by calculating the orientational autocorrelation function (OACF) <**u**(*t*)·**u**(0)>_L_ of the unit vector **u** directed along the chain end-to end vector (**R**_ee_), and its time decay in the course of equilibrium MD and NEMD runs. For ring melts, terminal relaxation is quantified by looking at the OACF <**u**(*t*)·**u**(0)>_R_ of the unit vector **u** directed along the diameter vector (**R**_d_) of the ring, averaged over all possible such vectors for a given ring molecule. The rate with which <**u**(*t*)·**u**(0)> approaches the zero value is a measure of how fast the chain forgets its initial configuration, i.e., of the rate of the overall orientational relaxation of the chain. 

**Figure 13:**
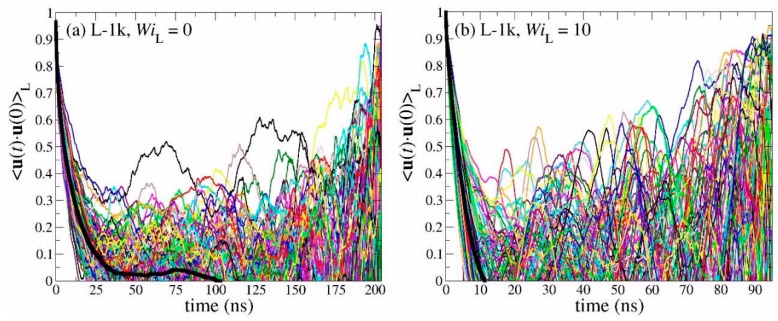

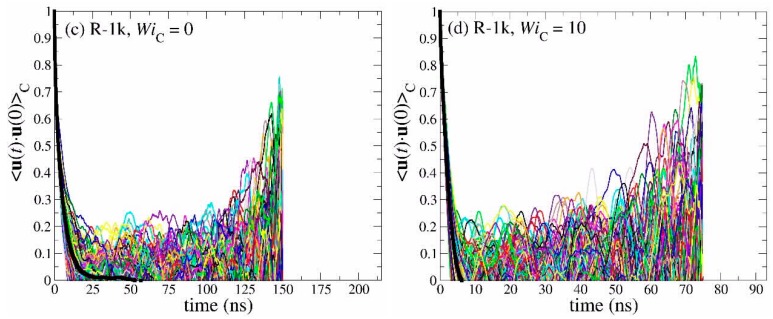
Decay of the time autocorrelation function <**u**(*t*)·**u**(0)> for each individual chain in: (**a**) the L-1k melt at equilibrium, (**b**) the L-1k melt under strong flow (${Wi}_{L}=10$), (**c**) the R-1k melt at equilibrium, and (**d**) the R-1k melt under strong flow (${Wi}_{C}=10$). The thick black lines represent the average over all chains.

**Figure 14:**
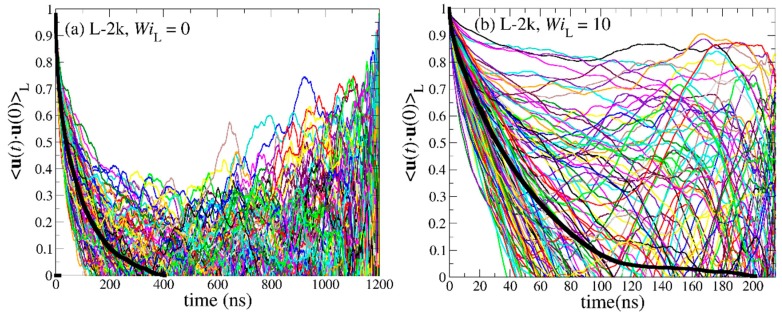

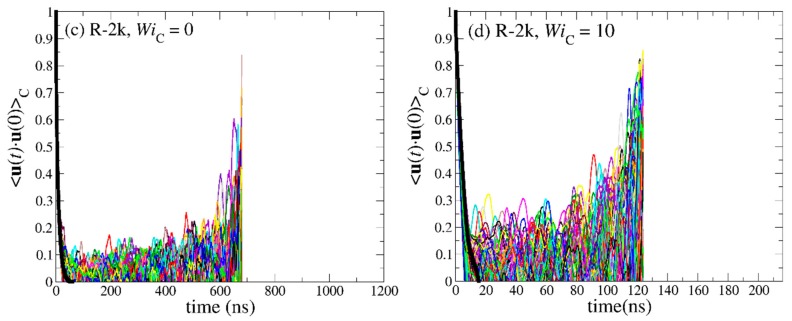
Same as with Figure 12 but for the PEO-2k system.

**Figure 15:**
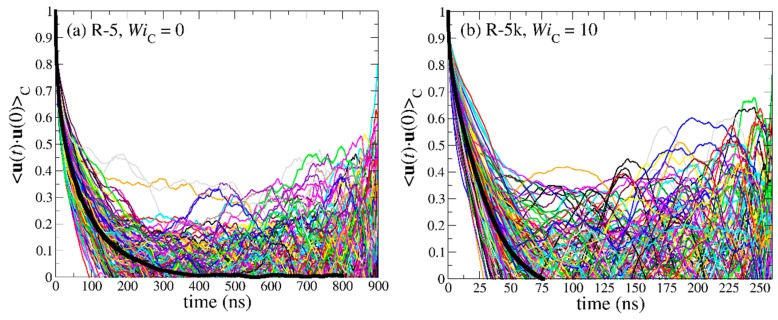
Decay of the time autocorrelation function <**u**(*t*)·**u**(0)> for each individual molecule in the R-5k melt: (**a**) at equilibrium, and (**b**) under flow (${Wi}_{C}=10$). The thick black lines represent the average over all chains.

Figure 13 shows the spectrum of the individual OACF functions separately for each chain in the simulated PEO-1k ring and linear melts, together with the corresponding average values (thick black lines), under equilibrium conditions and under strong shear flow corresponding to ${Wi}_{C}=10$ and ${Wi}_{L}=10$, respectively. The simulations have been carried out until the individual **u**(*t*)·**u**(0) functions for all chains in the melt have dropped to zero. Surprisingly, our MD simulations reveal that, for both molecular architectures studied (ring and linear), the orientational dynamics are highly heterogeneous, since the individual **u**(*t*)·**u**(0) curves deviate significantly from the average <**u**(*t*)·**u**(0)> curve. In fact, this dynamic heterogeneity is more pronounced in the case of linear melts where, in addition, chains require much longer times to relax. For the PEO-1k system examined in Figure 13, this behavior is counter-intuitive, since it is unentangled and, in addition, excess free volume around chain ends should accelerate chain relaxation in the case of the linear melt [12]. However, we can explain this peculiar behavior if we recall that, due to their looped structure, ring chains assume conformations that are spatially extended to shorter distances than chains in the linear melt. That is, terminal relaxation for linear chains involves the decorrelation of the unit vector along the end-to-end chain vector **R**_ee_ which corresponds to twice the molecular length covered by the diameter **R**_d_ vector used to define the OACF function in the case of rings. Under strong flow conditions (${Wi}_{C}=10$ and ${Wi}_{L}=10$), both types of melts relax faster but the main features of the decorrelation still remain. With increasing molecular weight (see Figure 14), the effect of excess free volume in the linear melts becomes less and less important, and this is reflected in the enhanced dynamic heterogeneity of linear chains. Similar behavior was observed for the entangled R-5k melt (see Figure 15).

**Figure 16:**
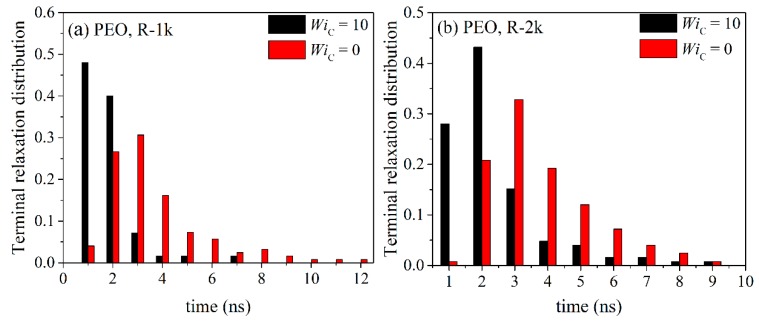

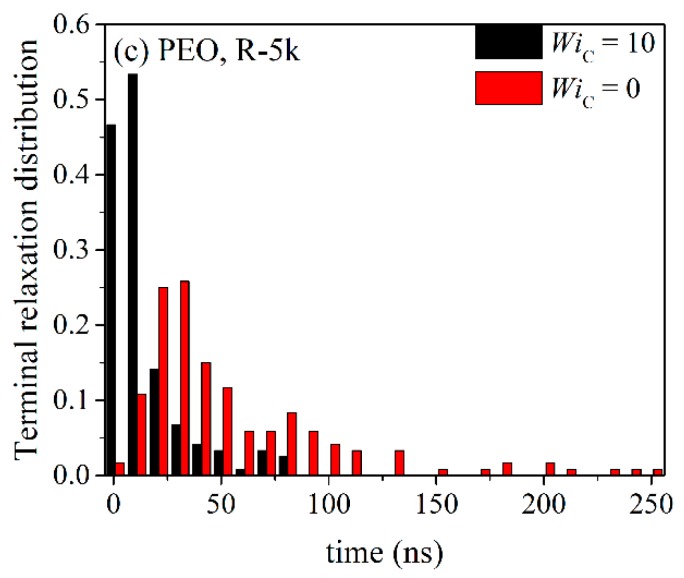
Histograms of the characteristic orientatonal relaxation times at equilibrium and under flow for the simulated: (**a**) R-1k, (**b**) R-2k and (**c**) R-5k ring PEO melts.

**Figure 17:**
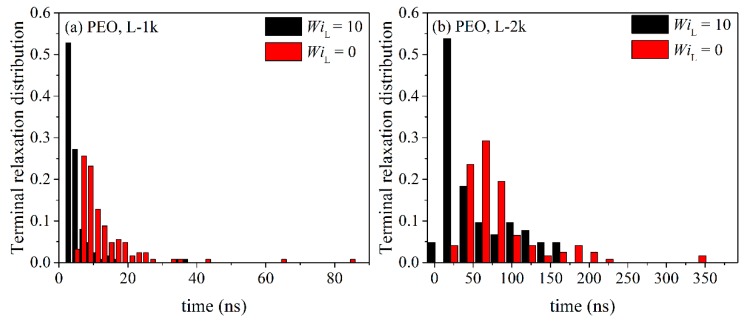
Same as with Figure 16 but for the two linear PEO melts.

By integrating the time autocorrelation functions <**u**(*t*)·**u**(0)>_R_ and <**u**(*t*)·**u**(0)>_L_ for all chains in the melt, one obtains a measure of the characteristic time constants for relaxation for ring and linear melts, respectively. The corresponding histograms of these times are displayed in Figure 16 and Figure 17. For all PEO systems studied, characteristic relaxation times for chains in the linear melt span a much wider range than in the ring melt. With increasing molecular length, the distributions for both types of melts become broader and, of course, their peaks are shifted to larger times. The applied flow has a strong effect on the distributions causing their width and average value to decrease.

### 3.4. Topological Analysis

As already mentioned several times so far in our article, the linear PEO-5k melt is weakly entangled, characterized by approximately *Z* = 2.5 entanglements per chain. On the other hand, several computational works published recently [4,5,39] have provided convincing evidence that in melts of ring molecules, strong ring–ring threading events develop as a ring molecule penetrates through other rings and, simultaneously, itself is threaded by other rings. In fact, the geometric analysis of Tsalikis et al. [5] demonstrated that, in many cases, these events involve many rings: for example, one ring can be threaded not by one but by several other rings, and vice versa. The same work also demonstrated that the majority of these ring–ring threading events are short-lived and thus have little influence on ring molecule dynamics. However, it also revealed that a non-negligible fraction of the events are long-lived, thus having a strong effect on ring dynamics. Indeed, accounting for these long-lived topological interactions in the *G*(*t*) of the ring melt in the same way as reptation theory accounts for entanglements in the *G*(*t*) of linear polymers could explain the long relaxation modes observed experimentally in melts of polymer rings [5].

It would therefore be of interest to analyze, even in a preliminary way, how the applied flow modifies the above picture. To do this, atomistic configurations from the simulated PEO melts under equilibrium and nonequilibrium conditions were subjected to a detailed topological analysis following the methodology described in earlier works [5,40], with the help of the CReTA algorithm of Tzoumanekas and Theodorou [41] which reduces atomistic configurations to an ensemble of primitive paths (PPs) in which chains are not allowed to cross each other as the algorithm simultaneously reduces the contour length of each chain. For linear melts, at the end of CReTA, kinks along the PP of each polymer chain are identified as points where a rectilinear segment of one chain meets a rectilinear segment of another chain. These kinks are considered as representing entanglement points in the melt, thus their number *Z*_k_ per chain is proportional to the number of underlying entanglements *Z* per chain. For ring melts, the same concepts apply but the subsequent identification of threading events between rings requires a more elaborate analyis based on 3D vector calculus. All details regarding the implementation of the CReTA algorithm for melts of polymer rings and the geometric analysis for identifying ring–ring threadings can be found elsewhere [5,40].

**Figure 18:**
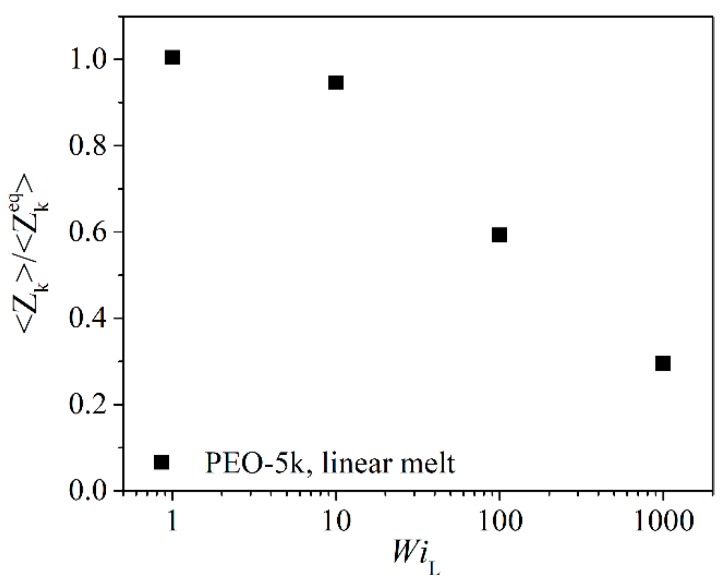
Number of kinks per chain normalized with the corresponding number at equilibrium for the L-5k PEO melt as a function of applied shear rate (in dimensionless units).

In Figure 18, we examine the effect of shear rate on kink number density for the L-5k melt. In particular, we report the average number of kinks per chain normalized with the corresponding value, $<Z_{k}^{\mathrm{eq}}>$, under equilibrium conditions. At low shear rates (${Wi}_{L}=1$), <*Z*_k_> remains practically unaffected by the flow. However, as the shear rate increases, chains orient in the direction of flow (see Figure 5a), which causes the average number of kink points per chain <*Z*_k_> to drop. This indicates a significant change of the underlying topological network with the applied flow, exactly as has been reported in other computational studies [32,42,43,44] for linear PE melts.

**Figure 19:**
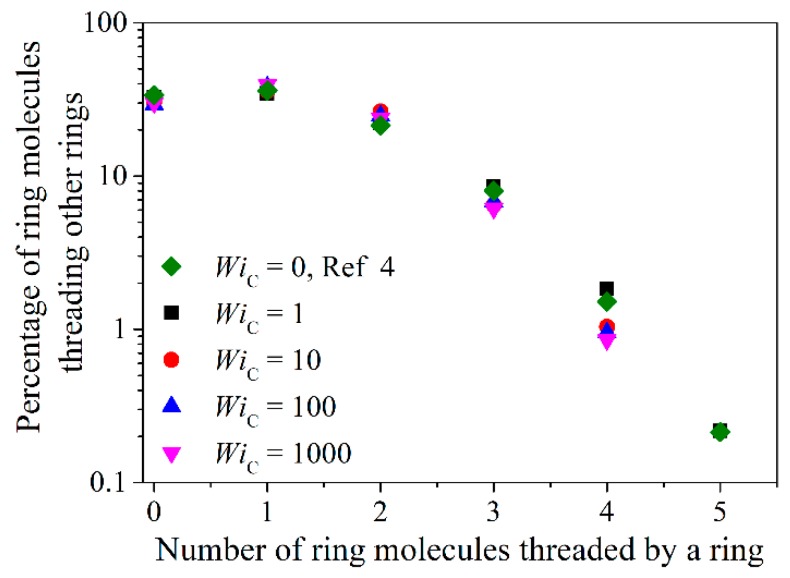
Histogram (in log-linear coordinates) of the number of ring–ring threadings in the R-5k melt for various shear rates.

**Figure 20:**
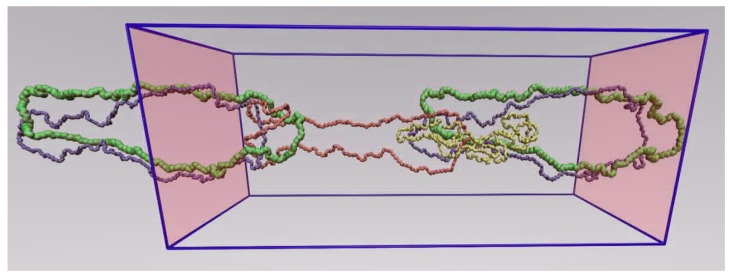
Example of a multiple threading event from the NEMD simulation with the R-5k melt under strong shear flow (${Wi}_{C}=1000$). The green ring molecule is simultaneously threaded by the blue, the orange and the yellow ring molecules. For clarity, we have included parts of the periodic images of the blue and green rings in the direction of flow.

Figure 19 presents the effect of shear rate on the statistics of threading events for the corresponding ring system, the R-5k melt. At small shear rates (${Wi}_{C}\leq 1$), the results are practically indistinguishable from the equilibrium ones. Surprisingly enough, the same holds also at higher shear rates (${Wi}_{C}$ = 10 or 100), except for some differences in the statistics of multiple threadings involving three or more rings. Given the large decrease in the number of entanglements per chain in the same regime of shear rates for the linear PEO-5k melt shown in Figure 18, this is a rather unexpected result. Even more surprising is that the same behavior is observed at extremely high shear rates (${Wi}_{C}=1000$) where rings are considerably deformed and aligned: the population of threading events seems to remain practically unaffected by the flow except for the statistics of multiple threadings which are seen to decrease considerably (or even to disappear completely). To explain this behavior, we calculated the survival (or disengagement) times of those threading events that were found to survive the longest under strong shear rates and their penetration length [4,5]. Interestingly enough, we found out that practically all of them are short-lived, and (even more importantly) that they are characterized by very short penetration lengths. More precisely, for shear rates corresponding to ${Wi}_{C}>10$, truly long-lived threadings were never observed. We also found that, in distinct contrast to the situation under equilibrium conditions where about 5.8% of threadings correspond to full penetrations [5], under flow conditions, full penetrations occur only occasionally. To make this message more clear, in Figure 20 we show a typical atomistic snapshot of a multi-threading event from the NEMD simulations with the R-5k melt at ${Wi}_{C}=1000$ where the ring with the green color is simultaneously threaded by three other rings shown in yellow, orange and blue. As we can see, in this multi-threading event only a small portion of the surfaces spanned by the three threading rings crosses through the surface of the threaded green chain. As one can understand, the topological analysis can reveal a wealth of information regarding the interplay between flow, topological interactions and rheological response of ring melts under flow which deserves a more detailed and thorough study in the future.

Our main explanation for the transition from the long survival times observed under equilibrium conditions to the short ones observed under flow conditions is ring chain deformation, which prevents other rings from penetrating it. According to our NEMD simulations, polymer rings under flow get significantly elongated in the direction of flow which, in conjunction with their closed loop structure, increases the degree of chain compactness, thus rendering it too difficult or too improbable for other rings to thread them. What we observe under flow conditions is only a very small degree of ring–ring inter-penetration, as a ring can get inside another ring only infinitesimally. As a result, and in clear contrast to the situation under equilibrium conditions, strong ring–ring threadings under shear flow disappear, implying a very minor or secondary role of the corresponding topological interactions on ring dynamics compared to other effects such as local intramolecular re-arrangements or large conformational fluctuations.

## 4. Conclusions

We have presented a comparative study of the steady state shear rheology between ring and linear polymer melts from detailed NEMD simulations using PEO as a model system. We have addressed three systems (PEO-1k, PEO-2k, PEO-5k) with molecular weights below and just above the characteristic entanglement molecular weight *M*_e_ of linear PEO. Our simulation results provide useful information concerning the effect of imposed shear rate on viscometric or material functions, conformational properties, terminal relaxation and topological interactions, and how these differentiate between ring and linear melts.

For the shortest, unentangled ring PEO melt examined (PEO-1k), the rheological response was very similar in the two melts (ring and linear), in excellent agreement with the experimental findings of Nam and co-workers [36]. With increasing chain length, the viscosity curve for the ring melt was found to fall below that for the linear and decline with shear rate at a smaller rate. We attributed this behavior to the lesser deformability of ring chains upon shearing due to their more compact structure as dictated by the absence of free ends. Similar observations have been reported in the literature by another NEMD simulation for PE [14] and experimental measurements [13].

It was also observed that ring melts follow Rouse dynamics for considerably longer lengths than linear melts; for the latter, excess free volume phenomena around chain ends at short molecular lengths and entanglement effects at long molecular lengths considerably shrink the regime of molecular weights for which the Rouse theory applies.

By analyzing terminal relaxation in the two types of melts for individual chains for long enough times (longer than the average orientational relaxation time), we observed that linear melts exhibit a stronger dynamic heterogeneity and molecular individualism than rings, in the sense that the corresponding histograms spread more around the average relaxation time. With increasing chain length, for both types of melts, the distribution of relaxation times becomes broader, with a clear tendency to shift to larger time scales. The application of the shear flow, on the other hand, was found to suppress the distribution of relaxation times. This was observed to be the case both for rings and linear melts.

A subsequent geometric analysis of topological constraints (entanglement kinks in the case of linear chains and threading events in the case of rings) in the marginally entangled PEO-5k showed that the underlying topological network remains unaffected in the regime of small shear rates corresponding to the Newtonian plateau (${Wi}_{L}{,Wi}_{C}<1$). For intermediate shear rates (${1<Wi}_{L}{,Wi}_{C}<100$), the entanglement density in the case of the linear melt was computed to reduce considerably due to chain deformation and alignment in the direction of flow. In striking contrast, the population of ring–ring threading events in the same regime in the case of the ring melt remained practically unaffected by the applied flow field. For very strong flows (${Wi}_{L}{,Wi}_{C}>100$), the entanglement density in the case of the linear melt dropped almost to zero. In the case of the ring, surprisingly enough again, the number of threading events reduced only marginally compared to that under equilibrium conditions, and this was mostly (or exclusively) for multi-threading events involving more than three rings. To explain this peculiar behavior, we carried out a detailed analysis of survival times of these threading events under shear and of their effective penetration length. We discovered that in (almost) all cases, all remaining threadings are short-lived, characterized also by very small penetration lengths, rendering them practically inactive (i.e., with a minimal effect on chain dynamics). In fact, no threading events corresponding to full penetrations were identified under strong flow conditions. We remind the reader that, under equilibrium conditions, Tsalikis et al. [5] had found that almost 5.8% of the identified ring–ring threading events corresponded to full penetrations.

The present work focused exclusively on the simulation of the steady-state shear rheology of pure ring melts and its comparison with that of the corresponding linear ones. In the future, we plan to extend our study in two directions. In the first, we will address the transient rheology of ring and linear PEO melts. In the second, we plan to investigate how ring melt rheology is affected by contamination in linear chains.

## Figures and Tables

**Table 1:** Some technical details (degree of polymerization, molecular weight, number of chains in the simulation cell, mean equilibrium radius of gyration, maximum diameter length and box length in the direction of flow) concerning all pure poly(ethylene oxide) (PEO) melts studied in this work.

System	*M*_w_ (g/mol)	Number of Molecules	$R_{g}^{\mathrm{eq}}\;(Å)$	$R_{d}^{\max}{,R}_{\mathrm{ee}}^{\max}\;(Å)$	*L*_x_ (nm)
R-1k	1320	3750	9 ± 1	54	187
R-2k	1844	3125	11 ± 1	74	177
R-5k	5324	1458	18 ± 1	220	207
L-1k	1322	3750	13.5 ± 1	108	193
L-2k	1846	3750	15.5 ± 1	148	202
L-5k	5326	1920	26 ± 1	440	295

**Table 2:** Nonequilibrium molecular dynamics (NEMD)-based zero-shear rate viscosities extracted by fitting the simulation data to the Carreau model, along with experimental data [19,33]. For comparison, we also show the viscosities as predicted by the Rouse model expressions for ring and linear polymer melts, with the ring and linear chain orientational relaxation times obtained from independent equilibrium molecular dynamics (MD) runs.

System	Carreau Model		Rouse Theory		Experimental Data	
	*η*_0,R_ (mPa·s)	*η*_0,L_ (mPa·s)	*η*_0,R_ (mPa·s)	*η*_0,L_ (mPa·s)	*η*_0,R_ (mPa·s)	*η*_0,L_ (mPa·s)
PEO-1k	16.8 ± 1	17.3 ± 1	19 ± 1	19 ± 0.5	-	-
PEO-2k	32 ± 3	66 ± 5	24 ± 1	50 ± 5	30.2 ± 2	62.7 ± 5
PEO-5k	96 ± 10	269 ± 12	65 ± 5	553 ± 20	122.3 ± 20	-

## Supplementary Materials

The following are available online at https://www.mdpi.com/2073-4360/11/7/1194/s1.
